# Modification of the Rosenberg Scale to Assess Self-Esteem in Children

**DOI:** 10.3389/fpubh.2021.655892

**Published:** 2021-06-17

**Authors:** Carly Wood, Murray Griffin, Jo Barton, Gavin Sandercock

**Affiliations:** School of Sport, Rehabilitation and Exercise Sciences, University of Essex, Colchester, United Kingdom

**Keywords:** global self-esteem, children, health, confirmatory factor analysis, factorial invariance

## Abstract

Rosenberg's scale (RSES) is widely used to assess global self-esteem (SE) in adults and adolescents but is not validated for children <12 years. This study assessed the internal consistency, convergent validity, and factor structure of a modified RSES for schoolchildren (CRSES) aged 7–12 years. A total of 711 children aged 9.0 ± 1.5 years completed the CRSES; a subset (*n* = 417) also completed a life satisfaction (LS) scale. Data were submitted for confirmatory factor analysis (CFA) and tests of factorial invariance by sex. Two-way ANOVA compared scores by age-group and sex; whilst Pearson's correlations examined the relationship between LS and SE. Following the use of modification indices the fit for the global SE model met the goodness of fit statistic criteria: χ(27, *n* = 711) = 77.22; χ^normed^ = 2.860 CFI = 0.961; RMSEA = 0.051 with 90% CI = 0.038-0.065; SRMR = 0.037; and displayed respectable internal consistency (α = 0.79). The model was also factorially invariant by sex. SE scores did not vary sex (*p* > 0.05); but were significantly reduced in children aged 9–10 and 11–12 years compared to children aged 7–8 years. The global SE score was significantly correlated (r = 0.51; *P* < 0.001) with LS. The current version of the CRSES can reliably examine global SE in children aged 7–12 years; extending the use of the RSES to allow tracking across the life course.

## Introduction

Self-esteem (SE) can be defined as *a person's positive or negative attitude toward the self in totality* (1). This valuable personal asset predicts better quality of life and personal empowerment (2, 3); low SE is a risk factor for anxiety and depression and has a prospective impact on real-world life outcomes (4–8). Orth et al. (9) found that low SE during childhood preceded negative life-course trajectories for: affect, depression, satisfaction with relationships and employment, and physical health. Low SE in young adults is associated with worse economic prospects and criminal behavior in later life (9–11). Such prospective associations suggest that SE might be a cause, rather than a consequence of life outcomes.

The study of SE across the lifespan requires psychometrically sound tools suitable for all ages. Scales producing comparable scores allow SE to be tracked longitudinally. As SE varies by age and sex (1, 12) interpretation of scores against population specific normative-values or criterion referenced cut-offs are also essential.

Rosenberg's SE Scale (RSES) was developed to describe, compare, and predict SE and evaluate changes in SE due to interventions (13). It is the most widely utilized, valid, and reliable measure of SE and has been extensively used in psychological research (14–16). In healthy adults the scale's single factor structure confirms its utility as a measure of global SE (17–19).

Bagley and Mallick (1) confirmed the factor structure and reliability of the RSES in adolescents aged 12–19 years; and produced normative data by reporting age specific means for males and females. They also provided evidence for convergent validity reporting positive correlations with validated measures of overall health. Validity of RSES has not been established in children younger than 12-years, possibly because the scale includes complex wording and abstract concepts that may not be easily understood by younger children. Rosenberg and Simmons (20) did develop and use a 6-item Guttman scale version of the RSES for individuals aged 8–18 years, which was subsequently used by Rosenberg and Pearlin (21) and Simmons et al. (22); however, this version of the scale was designed to be administered by interview, making it inappropriate for use with large sample sizes. A 7-item Guttman version of the scale was also used in a study of children and adolescents in New York (20); however the scoring method of both this version and the 6-item version contrast that of the original RSES; making it difficult to make longitudinal comparisons. Given the increasing prevalence of mental-ill health in children (23) and the possible impact of SE on life course trajectories (9); there is a need for valid and reliable measures of SE during childhood. The 10 item RSES is already used to assess SE in adults and adolescents. Developing a scale providing a broadly comparable measure of SE in younger children therefore appears prudent.

Our primary aim was, therefore, to assess internal consistency and factor structure of a modified RSES for measurement of SE in English schoolchildren aged 7–12 years. Based on research in adults and adolescents, we aimed to modify scale items to reflect a one-factor global SE measure. Our secondary aim was to provide reference values for this population by comparing with and extending the range of extant normative data (1). We hypothesized that scores of older children aged 11–12 years would approximate those reported previously (1) and be higher in younger children aged 7–8 and 9–10 years. We also hypothesized that SE would be lower in females, supporting previous evidence of sex differences (1). Finally, we aimed to assess the convergent validity in terms of correlation with life satisfaction (LS).

## Materials and Methods

### Questionnaire Development

The authors, who are experienced in using the RSES in research with adolescents and adults, examined the scale and highlighted any sections which they felt would not be understood by children as young as 7 years of age. The phraseology of the RSES was deemed too complex, but it was felt that if the language was simplified children would be able to understand the meaning of all items. For example, item one on the original scale (“I feel like I am a person of worth, at least on an equal plane with others”) was thought to be too complex for children to interpret; but it was felt that the meaning of the phrase could be understood if the terminology was simplified. This phrase was therefore altered to “I feel that I'm as good as everyone else.” The interview version of RSES developed by Rosenberg and Simmons (20) also maintained the meaning of the selected phrases but altered the wording to reflect terminology used by children; this included giving examples of feelings experienced by children and asking the child taking part in the interview if they felt this way and whether they did so a little or a lot. The authors therefore simplified any words or phrases which they felt were too difficult for children to understand (Appendix 1).

### Review by Experts

Following development by the authors, the child version of RSES (CRSES) scale was reviewed by two experts. Both experts had extensive experience in using the original scale as well as expertise in questionnaire development and validation. The experts were asked to independently examine the extent to which the items on CRSES could be understood by children and assessed their SE. The suggestions from experts were then incorporated into the updated scale. The main suggestion from the reviewers was to alter the response options; these were amended from “strongly agree,” “agree,” “disagree,” and “strongly disagree”; to “very true,” “true,” “not true,” and “definitely not true.”

### Participants

A total of 711 children (*n* = 382 males; 53.7%) aged 9.0 ± 1.5 years (mean ± SD; range 7–11 years) took part in the study; recruited through researcher links with schools. The sample represents 93.8% of available participants drawn from a sample stratified at school level. The school level stratification of sampling ensured that a representative proportion of participants from each of the five quintiles of area level deprivation, as defined in the UK based on postcode identification of dwelling, were included. Schools were purposefully recruited from within each of the five quintiles of area-level deprivation based on their index of multiple deprivation (IMD) (24). A stratified sub-sample of 417 children (*n* = 217 males; 52%) aged 8.3 ± 1.2 years (mean ± SD; range 7–11 years) also completed an additional questionnaire (see questionnaire completion section below). The only exclusions at participant level were students that did not normally participate in core Physical Education (PE) classes as defined in the UK Government national curriculum standards. This excluded children unable to access PE facilities (typically only those with physical disability requiring use of a wheelchair) and children with special educational needs severe enough to preclude their participation in core PE activities and classes.

### Questionnaire Completion and Scoring

All participants completed the CRSES on one occasion. Participants were asked to complete the questionnaire independently and honestly, based upon their feelings at that precise moment. Questionnaires were completed inside the school environment in the presence of the researchers and class teachers. In alignment with the original RSES, we included 10 items within the CRSES. The scale comprised five positive and five negative statements each with four possible responses scored from one to four. Scoring was reversed for responses to negative items so that a higher score indicated better overall SE; with scores ranging from 10 to 40, as per the data of Bagley and Mallick (1).

LS (*n* = 417) was also assessed in a sub-sample of participants, who were taking part in a wider research study (25). The LS scale consists of six items and requires the participant to identify how happy they are from 1 (completely happy) to 7 (not happy at all) with different aspects of their lives. A modified version of the scale was used whereby the family question was removed due to some schools feeling that it might be too upsetting. The score ranged from 5 to 35, with scores being inverted so that a higher score represented better LS. The Cronbach alpha for the current sample was α = 0.71.

### Statistical Analysis

#### Confirmatory Factor Analysis

Data were submitted for confirmatory factor analysis (CFA) using AMOS v23. The sample size provided a participant-to-item ratio of 71:1; in excess of the conservative requirement of a 10:1 ratio (26). The overall fit of the global SE model was assessed using a range of goodness of fit statistics. The normed Chi-square model was examined and represents the ratio of χ^2^ to degrees of freedom (called χ^normed^) (27). A value of 3.0 or less was used to indicate a good fit (28). The comparative Fit Index (CFI) was used to determine how well the model compared to a nested baseline model. A value close to or >0.95 indicates an adequate model fit and was therefore used as the cut off value. The Standardized Root Mean Square Residual (SRMR) assessed the mean absolute correlation residual, with a smaller SRMR indicating a better fit. The cut off of <0.08 recommended by Hu and Bentler (28) was used for this statistic. The Root Mean Square Error of Approximation (RMSEA) was examined to measure the extent to which the models were supported per degree of freedom. RMSEA values close to 0.06 indicate a good fit, with values ranging to 0.10 representing a mediocre fit (28). Factor loadings were interpreted using Comrey and Lee's (29) recommendations where 0.71 = excellent, >0.63 = very good, >0.55 = good, >0.45 = fair, and >0.32 = poor. Modification indices (MI) were also consulted to improve the accuracy of the model. A value of 10 was used to guide model refinement; with modifications to the model making theoretical and statistical sense.

Cronbach alpha was examined to assess the internal consistency of the model. A Cronbach alpha coefficient of between 0.65 and 0.70 is minimally acceptable, 0.70 to 0.80 respectable, and >0.80 very good (30).

#### Factorial Invariance

Tests of factorial invariance were conducted between males and females. Configural invariance was determined by examining the goodness of the model fit using a freely estimated model across the two groups. Metric invariance was examined by constraining the two models to be equal and conducting a chi-square test on the fully constrained and unconstrained models. Finally, scalar invariance was evaluated where all item-factor intercepts were constrained equally across sex and evaluated against the factor loading invariance model. *P* > 0.05 indicates factorial invariance. Cronbach alpha was examined to assess the internal consistency of the model in males and females separately.

#### Normative Data and Content Validity

Mean data were generated for males and females according to three different age groups: (i) 7–8 years; (ii) 9–10 years; and (iii) 11–12 years; and tabulated alongside the normative data of Bagley and Mallick (1). Two way between subject's ANOVA was used to compare SE across the three different age groups according to sex; with *post-hoc* Tukey being used to compare scores between the three age groups. Finally, Pearson's correlation was used to examine the relationship between CRSES and LS scores.

## Results

### Confirmatory Factor Analysis

**Figure 1:**
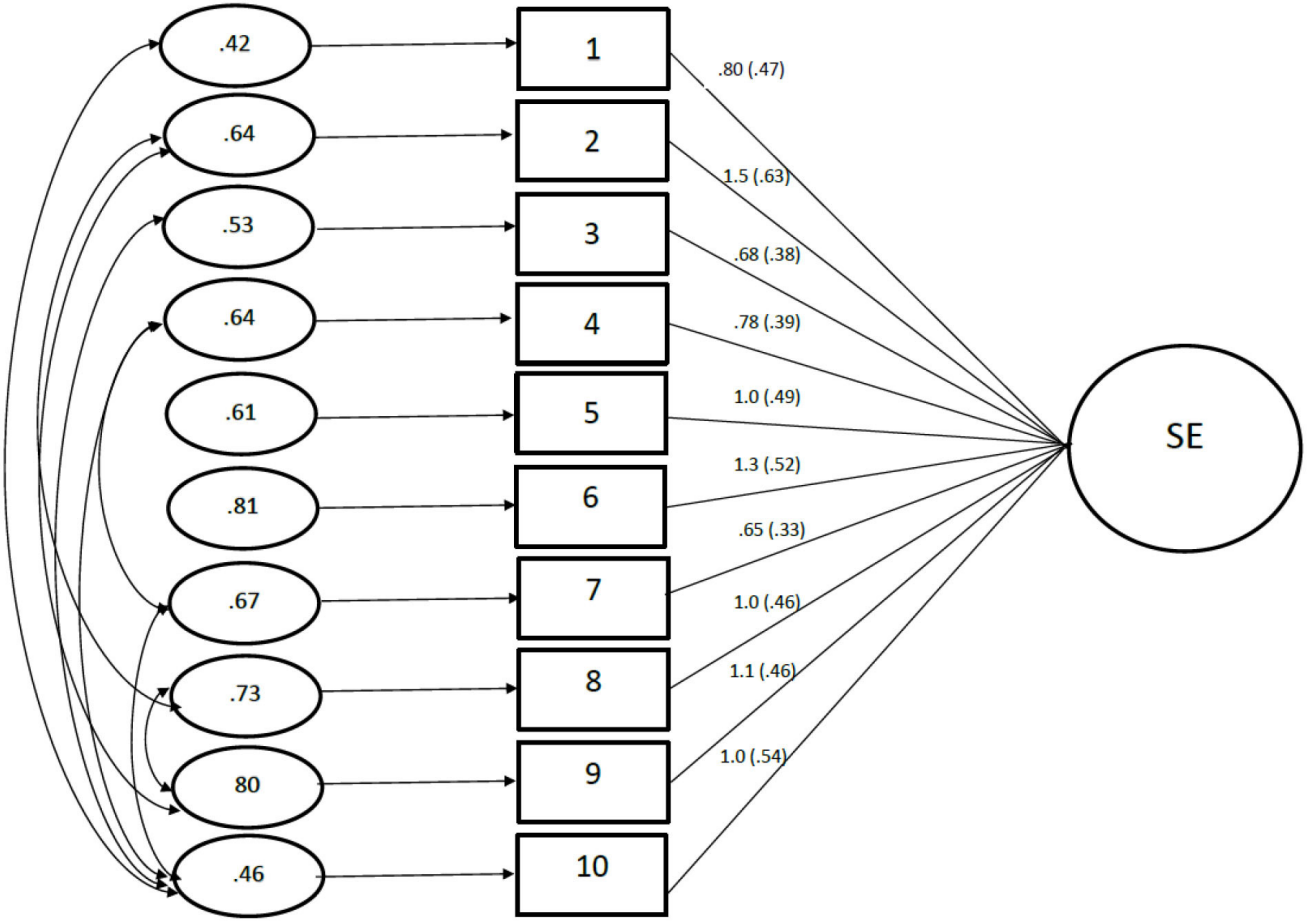
Global SE model for the Child Rosenberg Self-esteem Scale. Item numbers in the figure represent the items in the Child Rosenberg Self-esteem Scale. The large circle is the global self-esteem score, with the rectangles representing the measured variables, and the small circles with numbers are variances. The factor loadings are standardized in parenthesizes and unstandardized outside.

Fit indices for the global SE model were: χ(36, *n* = 711) = 296.22; χ^normed^ = 8.463; CFI = 0.799; RMSEA = 0.103 with 90% CI = 0.092–0.113; SRMR = 0.074. The value for the SRMR was within recommended values; whilst the RMSEA represented a mediocre fit. However, the χ^normed^ was greater than the recommended value of 3.0; and the CFI was <0.95, indicating poor model fit. Modification indices were consulted to free error covariance between items 4–7, 1–10, 3–10, 4–10, and 7–10; which represented positively worded items; and items 2–8, 2–9, 8–9, which represented negatively worded items. The modification model met all Goodness of Fit criteria: χ(27, *n* = 711) = 80.820; χ^normed^ = 2.993 CFI = 0.959; RMSEA = 0.053 with 90% CI = 0.040–0.066; SRMR = 0.0377. All items had at least fair factor loadings on the global SE factor; with all factor loadings being significant (*p* < 0.001) and in the expected direction (Figure 1). The global SE model displayed respectable internal consistency (α = 0.76).

### Factorial Invariance

The global SE model was used as the baseline model for tests of factorial invariance. The unconstrained model had adequate fit for both males and females individually, suggesting configural invariance (see Table 1). Differences between the unconstrained and fully constrained model were not significant, indicating that the model achieved metric invariance across sexes [Δχ^2^_(9)_ = 3.548, *P* = 0.939]. Tests for scalar invariance also demonstrated invariance between sexes [Δχ^2^_(19)_ = 16.828, *P* = 0.602]. For both males (α = 0.79) and females (α = 0.74) the global SE score demonstrated respectable internal consistency.

**Table 1:** Model fit indices and tests of measurement invariance for the global SE factor across sex.

**Model**	**χ^**2**^**	***Df***	**${\text{χ}}_{\text{normed}}^{2}$**	**RMSEA (90% CI)**	**SRMR**	**CFI**
Boys (*n* = 382)	52.048	27	1.928	0.049 (0.029–0.069)	0.040	0.968
Girls (*n* = 329)	43.061	27	1.595	0.043 (0.015–0.066)	0.040	0.969
Configural invariance	96.607	54	1.789	0.033 (0.022–0.044)	0.034	0.967
Metric invariance	100.154	63	1.590	0.029 (0.018–0.039)	0.036	0.971
Scalar invariance	113.435	73	1.554	0.028 (0.017–0.038)	0.036	0.969

*RMSEA, Root Mean Square Error of Approximation; SRMR, Standardized Root Mean Square Residual; CFI, Comparative Fit Index*.

### Normative Data

Mean SE scores for the children in current study are displayed in Table 2 alongside the adolescent normative data of Bagley and Mallick (1). For the current sample, two-way ANOVA revealed no significant effect of the interaction of age and sex (*P* > 0.05; ${\text{n}}_{\text{p}}^{2}$ = 0.005) on SE and no significant differences in SE between males and females (*P* > 0.05; ${\text{n}}_{\text{p}}^{2}$ = 0.000). However, there was a significant main effect of age category [*F*_(2, 711)_ = 20.32; *P* < 0.001; ${\text{n}}_{\text{p}}^{2}$ = 0.055]; with a significantly reduced SE in individuals aged 9–10 years (*p* < 0.001) and 11–12 years (*p* < 0.001) compared to individuals aged 7–8 years. These differences were also significant when considering male and female data separately. SE scores for individuals aged 9–10 and 11–12 years are similar to scores produced by Bagley and Mallick (1). Pearson's correlation also revealed a significant positive relationship between the global SE score and LS (r = 0.51; *P* < 0.001); indicating a higher SE is associated with greater LS.

**Table 2:** Normative data in males and females.

	**Current data**				**Bagley and Mallick** **(****1****)**			
	**Age (yrs)**	**7–8**	**9–10**	**11–12**	**12–13**	**14–15**	**16–17**	**18–19**
Male	Mean (95% CI)	31.78 (31.00–32.56)	28.65 (27.82–29.48)	29.63 (28.55–30.71)	30.18	31.16	30.54	31.68
	SD	5.02	5.34	5.03	5.67	5.44	5.72	5.67
	%low SE	1.9	7.9	4.8	5.8	3.7	4.1	2.7
Female	Mean (95% CI)	31.35 (30.56–32.13)	29.59 (28.69–30.49)	28.75 (27.34–30.11)	28.51	27.86	28.37	28.84
	SD	4.85	5.06	4.32	5.49	5.36	5.36	5.47
	%low SE	1.9	5.0	3.8	4.8	7.8	5.7	4.7

*Low Self-Esteem is defined as a score <21 (1)*.

## Discussion

SE is important for a successful and satisfying life and constitutes a fundamental aspect of psychological well-being (9, 10, 31). Improving SE is a targeted outcome and key performance indicator of multiple public health interventions and policies (32). Valid tools are therefore required to assess and monitor change across the life course. We aimed to produce a measure of SE suitable for children <12 years of age by modifying RSES; a valid measure of SE used extensively in adult and adolescent populations.

The results of the CFA revealed that the CRSES provided adequate fit for the global SE factor; meeting criteria on all goodness of fit statistics and displaying respectable reliability. The model was also factorially invariant and reliable in males and females, indicating adequate fit in the two groups separately. These findings indicate that the scale can reliably be used to assess global SE in children aged 7–12 years and therefore further expands the use of RSES to enable tracking of SE across the life course. Given the importance of SE for life outcomes such as depression, relationship and job satisfaction and health (8–10); the ability to track and compare SE from childhood into adulthood using one scale with an analogous scoring mechanism is of great benefit. Furthermore, the CRSES developed in the current study has been used to monitor childhood SE in several published intervention studies (33–35).

The mean data for males and females generated in this study extends the published normative data for adolescents (1). However, unlike the normative data of Bagley and Mallick (1), there were no significant differences in SE scores between male and females; overall or within each age group. Previous research has indicated that sex differences in SE do not develop until adolescence; when the differences between males and females become more apparent due to puberty and development (36). Although it is difficult to identify the exact age at which adolescence occurs due to individual differences; the lack of sex differences in the current data might reflect that most participants were still in the period of childhood.

Our mean data also identified significant reductions in SE with increasing age, as per our hypothesis. The youngest age group (7–8 years) had significantly better SE than individuals in both the 9–10 and 11–12 years age groups. There were no differences in SE between individuals in the two older age groups (9–10 and 11–12 years). Previous research has identified that for both males and females; SE is typically higher during childhood, drops during adolescence, and then gradually rises into adulthood (12, 21). Our data, alongside that of Bagley and Mallick (1) supports this notion; with the youngest group having the best SE and a trend toward increasing SE upon entry to adulthood Whilst the significant reductions in SE between the 7–8 and 9–10 years were not necessarily expected, the reductions might reflect the beginning of the decline in SE that occurs into and throughout adolescence (12, 21).

The scores achieved on the CRSES were also correlated with scores for LS, indicating that children with better SE were more satisfied with their lives. These findings support the validity of the scale and are in line with previous evidence that has consistently identified relationships between SE, LS and health outcomes (1, 2, 9–11, 31).

A limitation of the current study is the lack of comparison of the scores achieved on the CRSES to other child measures of SE. Whilst it is interesting to consider the notion of determining the construct validity of the CRSES by comparing the data to existing child measures of SE, the lack of extensively used and reliable measures of SE for children make this is an inherently difficult enterprise. Researchers might compare the scores achieved on the interview version of RSES used by Rosenberg and Simmons (20), but as previously stated the demand characteristics of this approach are palpable. For example, children sometimes answer questionnaires based on factors such as their mood at the time of administration; or what they feel the researchers, or their teacher would like them to say. This being the case one might be guilty of not comparing like with like and the interpretation of the scores would need to be carefully considered, for example by exploring children's reasons for any putative discrepancies.

The study would also be improved by further examination of the relationship of scores on the CRSES with other health markers known to be correlated with SE, such as well-being and physical activity. Previous studies using the CRSES have identified a significant increase in SE due to physical activity interventions; in line with research documenting the positive benefits of physical activity for SE (37, 38). Additional data exploring these relationships would help to further support the convergent validity of the scale.

Overall, the findings of the current study indicate that the current version of the CRSES can be used to assess SE in children aged 7–12 years through calculation of a global SE score. The use of the CRSES and the provision of normative childhood data further extends the capabilities of the original RSES and allows the tracking of SE in children as young as 7 years of age, through to adolescence and adulthood. Given the possible impact of SE on life course health and behavioral outcomes (9) and its importance for psychology, public health, and social science research (32); the availability of this scale enhances the capacity for SE research in children.

## Data Availability Statement

The datasets presented in this study can be found in online repositories. The names of the repository/repositories and accession number(s) can be found at: University of Essex Research data Repository: 10.5526/ERDR-00000126.

## Ethics Statement

The studies involving human participants were reviewed and approved by University of Essex Ethics Committee. Written informed consent to participate in this study was provided by the participants' legal guardian/next of kin.

## Author Contributions

CW contributed conception and design of the study, performed statistical analysis, and wrote the first draft of the manuscript. GS contributed conception and design of the study, collected the data, and advised on statistical analysis. MG and JB contributed to study design and advised on statistical analysis. All authors contributed to manuscript revision and approved the final manuscript.

## Conflict of Interest

The authors declare that the research was conducted in the absence of any commercial or financial relationships that could be construed as a potential conflict of interest.
