# Supplementary material for: Modification of the Rosenberg Scale to Assess Self-Esteem in Children
Source: Front Public Health. 2021 Jun 17;9:655892. doi: 10.3389/fpubh.2021.655892 (PMC8247758; doi:10.3389/fpubh.2021.655892)
Supplement: Supplementary file 1 [file Table_1.DOCX]

**Appendices**

**Appendix 1: Child Rosenberg Self-esteem Scale**

|  | **Very True** | **True** | **Not True** | **Definitely Not True** |
| --- | --- | --- | --- | --- |
| I am happy with myself |  |  |  |  |
| Sometimes I think I’m no good at all |  |  |  |  |
| There are lots of good things about me |  |  |  |  |
| I can do things as well as most other children |  |  |  |  |
| I don’t have much to be proud of |  |  |  |  |
| I feel useless at times |  |  |  |  |
| I feel that I’m as good as everyone else |  |  |  |  |
| I wish I cared about myself more |  |  |  |  |
| I often feel like a failure |  |  |  |  |
| I feel good about myself |  |  |  |  |

**Adapted from Rosenberg (1965).**
